# Antimicrobial resistance in bacterial pathogens among hospitalized children with community acquired lower respiratory tract infections in Dongguan, China (2011–2016)

**DOI:** 10.1186/s12879-017-2710-4

**Published:** 2017-09-11

**Authors:** Xiaoguang He, Mingyu Xie, Siping Li, Junqin Ye, Qi Peng, Qiang Ma, Xiaomei Lu, Baimao Zhong

**Affiliations:** 1 Department of Pediatric Respiratory Medicine, Dongguan Children’s Hospital, Xihu Third Road NO. 68, Dongguan, Guangdong 523325 China; 2 Department of Pathogenic Microorganism, Dongguan Institute of Pediatrics, Dongguan, Guangdong China; 3 Dongguan Key Laboratory of Pediatric Genetic and Infectious Diseases, Dongguan, Guangdong China

## Abstract

**Background:**

Bacterial pathogens are a major cause of childhood community acquired lower respiratory tract infections (CA-LRTIs), and few data described the impact of antimicrobial resistance on children with CA-LRTIs. This study aims to investigate the antimicrobial resistance in common bacterial agents among hospitalized children with CA-LRTIs between 2011 and 2016 in Dongguan, China.

**Methods:**

Sputum samples were collected from hospitalized children (0–5 years old) with CA-LRTIs in Dongguan Children’s Hospital. Bacterial pathogens were detected using traditional culture methods, and disc diffusion tests were used to determine antibiotic resistance.

**Results:**

Among the 2360 samples analyzed, 342 (14.5%) were positive for bacterial infection. The most prevalent pathogen was MSSA (2.3%), followed by MRSA (1.5%), *E. coli* (1.7%), *E. coli* ESBLs (1.2%), *K. pneumonia* ESBLs (1.5%), *K. pneumonia* (1.4%) and *S. pneumonia* (1.3%). Of the hospitalized patients with bacteria causing of CA-LRTIs, 90.1% were less than 1-year-old. MSSA and MRSA were more commonly isolated in infants less than 3 months. *E. coli, K. pneumonia* and *K. pneumonia ESBLs* were more common bacteria causing CA-LRTIs in infants less than 1 month. Resistance levels to penicillins, fluoroquinolones, macrolides, cephalosporins, carbapenems and vancomycin varied in different bacteria.

**Conclusions:**

*S. aureus*, *E coli* and *K. pneumonia* were the common bacterial isolates recovered from chidren with CA-LTRIs during 2011–2015. Age group of under 1 year old was at a high risk of bacterial infections. Many isolates showed antibiotic resistance level was associated with antibiotic usage in clinic. Increasing surveillance of antibiotic resistance is urgently needed and develops better strategies to cure the antibiotic abuse in China.

## Background

Community acquired lower respiratory tract infections (CA-LRTIs) continues to be one of the most frequent infectious diseases causes of hospitalization and death worldwide, especially in children less than five years old [1–4]. Although respiratory viruses,such as respiratory syncytial virus, adenovirus and human metapneumovirus are significantly implicated in LRTIs at present, bacterial pathogens remain a major cause of CA-LRTIs in children, particularly in developing areas [5–7]. Among several previous Chinese studies, Qin et al. [8] found *Streptococcus pneumoniae* and *Haemophilus influenzae* were the predominant pathogens in Nanjing. Pei et al. [9] reported *S. pneumoniae* and *Escherichia coli* were the major bacterial agents in Xinxiang. Peng et al. [10] found *S. pneumoniae* and *Hemophilus parainfluenzae* were the main pathogens in Chongqing. The attributable fractions of each of these pathogens varied largely depending on geographical locations. However, limited information about bacterial pathogens trigging CA-LRTIs were reported in Dongguan, Southern China.

Antibiotic resistance is considered to be a worldwide problem, and unwise use of antibiotics has been recognized as a key contributor to the increasing rates of resistance [11]. Therefore, clinical practice typically uses empirical antibiotic selection to target the most likely pathogens based on antibiotic sensitivity data [12]. The antibiotic resistance associated with CA-LRTIs varies significantly depends on geographical locations and investigated populations [13, 14]. Therefore, it is not adequate to simply copy the existing guidelines from other countries, which may be inappropriate and lead to serious problems in clinical practice. For example, the incidence of aminoglycosides and quinolones resistant-MRSA is relatively high in the USA [15] and Thailand [16]. Penicillin-resistant *S. pneumoniae* is also relatively high in USA [17] and Southeast Asia [18]. Moreover, most studies have described patterns in the resistance of bacterial pathogens among adults with respiratory tract infections, these patterns may occur differently in adults and children [14, 19]. Dongguan is an industrial city in southern China, which has more than 10 million of people. Limited information regarding antimicrobial resistance patterns in Dongguan is available, and it is necessary to clarify the epidemiology and antibiotic resistance in this local area. The objectives of our study were to identify antimicrobial resistance in microorganisms in children diagnosed with CA-LRTIs in Dongguan, China; to provide guidance to local clinical pediatricians regarding the choice of appropriate antibiotic therapy and decrease the incidence of antimicrobial resistance.

## Methods

### Study population

This retrospective study was conducted at the Department of Pediatric Infectious Disease in Dongguan Children’s Hospital, which is a tertiary care center with over 300 beds, including Department of Pediatric Intensive Care Unit, Department of Neonatal Intensive Care Unit, Department of Pediatric Surgery, Department of Pediatric Orthopaedic and several key departments of common pediatric internal medicine, the daily outpatient visits are more than 1000.

During the period from March 2011 to June 2016, the hospitalized patients less than 18 years old displaying symptoms of CA-LRTIs were enrolled in this study if they met three inclusion criteria: 1) one or more respiratory symptoms, including cough, expectoration, dyspnea, pleuritic pain, or/and fever; 2) radiographic findings indicating evidence of pneumonia/bronchitis, such as a chest X-ray or computed tomography (CT) scan interpreted by an attending radiologist as showing pulmonary consolidation, opacity, or infiltrate; and 3) children suffering lower respiratory tract infections before admission. The exclusion criteria are: 1) Immunodeficiency disease; and 2) known *Mycoplasma pneumoniae* or *Chlamydia pneumoniae* infections, in that *Mycoplasma pneumoniae* antibodies were detected using Passive Particle Agglutination (SERODIA®-MYCO II, Japan), and lgG/M antibody to *Chlamydia pneumoniae* was detected using Colloidal (China) in our hospital.

### Microbiological methods

All the pediatric patients involved were asked to supply single sputum samples from lower respiratory tract for 1-2 ml before initiation of antibiotic therapy. Regrettably, blood samples were not obtained in the retrospective study. As obtaining meaningful sputum specimens were extremely difficult in babies, the clinical specimens in our research from lower respiratory tract were collected using sterility sputum aspirating tubes by trained personnel following standard operating procedures. All samples had been transported to the laboratory for processing and analysis under the conditions of ≤2 h and 4 °C. Each specimen must be in accordance with the microscopic quality criteria: squamous epithelial cells (SEC)<10 and white blood cells (WBC)>25, or SEC/WBC <1:2.5. 10ul sputum sample was streaked on MacConkey, blood and chocolate agar, respectively. MacConkey and blood agar were cultured under aerobic conditions in 35 ± 2 °C for 16–24 h. The chocolate agar was cultured under aerobic conditions in a 5% CO2 at 35 ± 2 °C for 16–24 h according to the guidance. Suspicious colonies were then subcultured on Tryptic Soy Agar using streak plat method for purification, preserved on semi-solid agar slants and stored in the refrigerator (with freezing temperature of about 4 °C) for subsequent analysis. The bacteria were identified by ATB Expression (BioMerieux, France) that is automatic detection machine. *Pseudomonas aeruginosa* ACTT 27853, *Escherichia coli* ATCC25922 and *Staphylococcus aureus* ATCC25923 were the reference strains of positive control. All the operations were in accordance with the manual.

Antibacterial susceptibility testing was performed using a semiquantitative agar diffusion test. We adjusted the turbidity of clinical cultures with sterile saline or broth to achieve a turbidity equivalent to a 0.5 McFarland standard, and then spread on Mueller-Hinton (MH) agar plate (Oxoid, USA). Dics should be applied to the MH agar plate within 15 min of inoculation. After incubation measure the diameters of zones of complete inhibition to the nearest mm. Zone margins should be read as the area showing no obvious growth detected by the unaided eye. Isolates were categorized as susceptible, intermediate, or resistant to each antibiotic according to CLSI classifications. Extended-spectrum beta-lactamases production, methicillin-sensitive *Staphylococcus aureus* and carbapenem-resistant *Pseudomonas aeruginosa* were identified using CLSI criteria (CLSI: http://clsi.org) (The 2011–2016 year of CLSI guidelines, the CLSI guidelines was renewed annually, and carried out according to the latest guidance.). Quality control was carried out using *Escherichia coli* ATCC25922, *Staphylococcus aureus* ATCC 29213, *Klebsiella pneumonia* ACTT 700603, *Pseudomonas aeruginosa* ACTT 27853, *S. pneumonia* 49,619, *Enterobacter* ATCC 35218. Our laboratory is under the supervision of Guangdong Clinical Test Center (a professional QS control organization in Guangdong province), the microbiological laboratory quality assurance was carried out three times a year with accordance to CLSI guidelines (CLSI: http://clsi.org).

### Data collection

Basic demographic data, including age, gender, date of hospitalization and investigation results, were recorded at bedside by a clinician. All laboratory data were recorded in the hospital computer data system.

### Statistical analysis

Statistical analysis was performed using the statistical software of SPSS (version 22; SPSS Inc., Chicago, IL, USA). Chi-squared and Fisher’s exact tests were used to compare categorical data. All tests were two-tailed, and a *p* value <0.005 was considered to be statistically significant (adjusting test level “a’ = 0.005″).

## Results

### Detection and distribution of bacteria

**Fig. 1:**
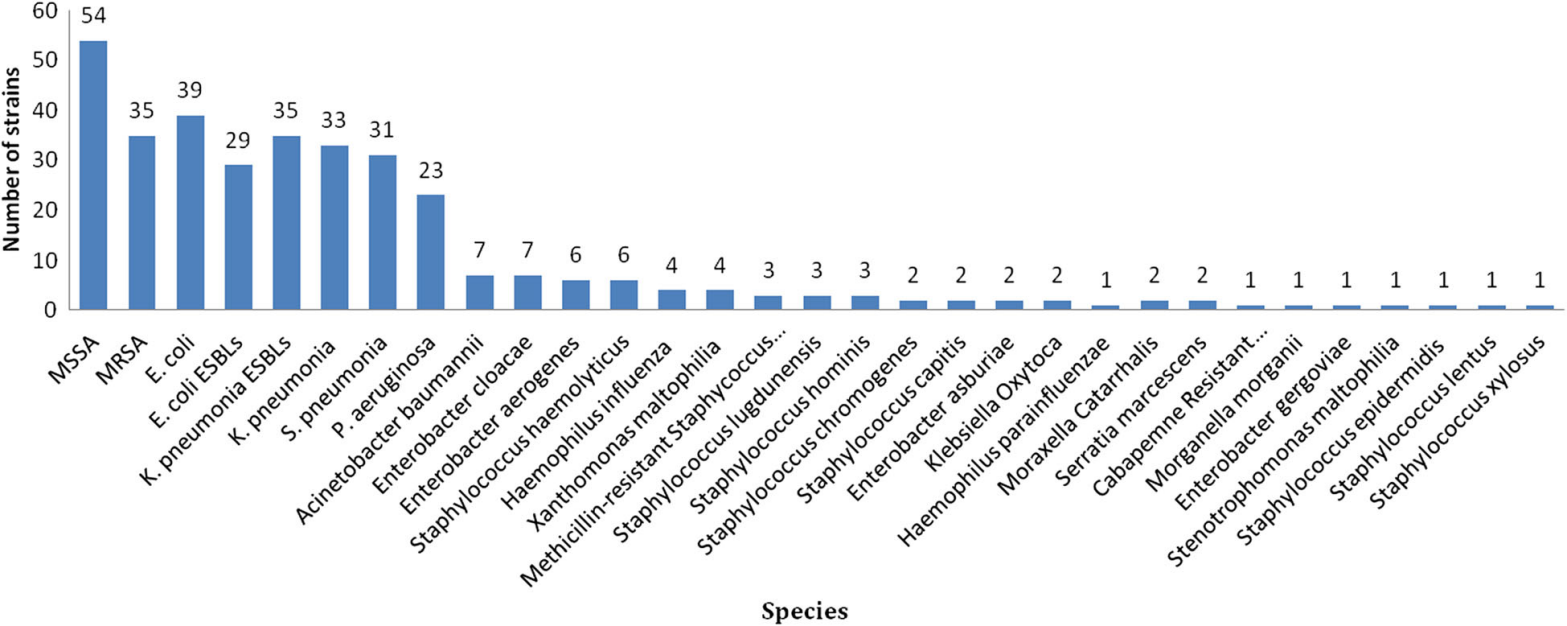
242 bacterial pathogens from 2360children with CA-LRTIs

From March 6, 2011 to June 5, 2016, a total of 2360 hospitalized pediatric patients met the inclusion criteria, including 1245 males and 1115 females; the mean age of these patients was 7.4 months (standard deviation: 10.3 months; range 0–5 years). Of the patients with CA-LRTIs, the majority were younger than 1 year-old (83.1%). 342(14.5%) bacteria were indentified, and the most frequent pathogen was Methicillin Sensitive *Staphylococcus aureus* (MSSA) (2.3%), followed by *Escherichia coli (E. coli)* (1.7%), Methicillin Resistant *Staphylococcus aureus* (MRSA) (1.5%), *Klebsiella pneumonia Extended Spectyum β-Lactamase* (*K. pneumonia ESBLs*) (1.5%), *K. pneumonia* (1.4%, *Streptococcus pneumoniae* (*S. pneumonia*) (1.3%), *Escherichia coli Extended Spectyum β-Lactamase (E. coli ESBLs)* (1.2%), *Pseudomonas aeruginosa* (*P. aeruginosa*) (1.0%), and other pathogens (2.7%) (Fig. 1).

In this study, all hospitalized patients with CA-LTRIs were divided into five age groups (Table 1). In the study, we found that infants (less than 1 month) were more common population with bacteria agents causing CA-LRTIs.Our results also showed that MSSA and MRSA were more commonly isolated in infants less than 3 months than that of other three age groups (P<0.005). *E. coli, K. pneumonia* and *K. pneumonia ESBLs* were more common bacteria causing CA-LRTIs in infants less than 1 month than that of other four age groups (P<0.005). However, our results indicated that *P. aeruginosa*, *E. coli ESBLs* and *S. pneumonia* infections had no significant difference in the five age groups.

**Table 1:** Distributions of common bacterial pathogens in age groups

Age	MSSA *n* = 54 (%)	MRSA *n* = 35 (%)	*E. coli n* = 39 (%)	*E. Coli* ESBLs *n* = 29 (%)	*K. pneumonia n* = 33 (%)	*K. pneumonia* ESBLs n = 35 (%)	*S. pneumonia n* = 31 (%)	*P. aeruginosa n* = 23 (%)	Others *n* = 63 (%)	Total *n* = 342 (%)
<1 m(*n* = 110)	9 (8.2)	7 (6.3)	8 (7.3)	1 (0.9)	12 (10.9)	9 (8.2)	1 (0.9)	4 (4.1)	22 (20.0)	73 (66.3)
1-3 m(*n* = 828)	36 (4.3)	22 (2.7)	18 (2.2)	13 (1.6)	12 (1.4)	16 (1.9)	7 (0.8)	5 (0.7)	21 (2.5)	150 (18.1)
4-6 m(*n* = 546)	6 (1.1)	3 (0.5)	8 (1.5)	11 (2.0)	8 (1.5)	10 (1.8)	12 (2.2)	3 (0.5)	13 (2.2)	74 (13.6)
7-12 m(*n* = 477)	1 (0.2)	1 (0.2)	3 (0.6)	3 (0.6)	1 (0.2)	0	6 (1.3)	2 (0.4)	4 (0.8)	21 (4.4)
1-5y(*n* = 399)	2 (0.5)	2 (0.5)	2 (0.5)	1 (0.3)	0	0	5 (1.3)	9 (2.3)	3 (0.8)	24 (6.0)
P value	<0.001	<0.001	<0.001	0.084	<0.001	<0.001	0.303	0.007	<0.001	<0.001

### Antimicrobial resistance

Table 2 illustrats the antimicrobial resistance of the most frequent microorganisms to penicillins, fluoroquinolones, macrolides, cephalosporin, carbapenems and vancomycin during 2011–2016. MSSA showed high resistance level of 81.5% to penicillin. All MSSA were sensitive to cefazolin in the study. Tested MRSA showed higher resistance to macrolides than MSSA, including erythromycin (81.8% vs. 29.6%), azithromycin (83.3% vs. 22.2%) and clarithromycin (82.8% vs. 34.5%). Noteworthly, MSSA exhibited higher resistance level to ofloxacin than MRSA (61.2% vs. 6.5%). Isolates of MSSA and MRSA exhibited similarity low resistance rates to gentamicin (4.2% and 9.4%, respectively) and co-trimoxazole (4.0% and 13.3%, respectively).

**Table 2:** Resistance rates of main bacteria pathogens causing pediatric patients with CA-LRTIs to antimicrobial agents during 2011–2016 in Dongguan

Antimicrobial agent	MSSA	MRSA	*E. coli*	*E. coli* ESBLs	*K. pneumonia*	*K. pneumonia* ESBLs	*S. pneumonia*	*P. aeruginosa*
Penicillin (10 U)	81.5%	100%	–	–	–	–	29.0%	–
Piperacillin (100μg)	–	–	59.0%	91.7%	24.2%	100%	–	21.7%
Ampicillin (10μg)	–	–	71.4%	100%	100%	100%	–	–
Erythromycin (15μg)	29.6%	81.8%	–	–	–	–	96.7%	–
Azithromycin (15μg)	22.2%	83.3%	–	–	–	–	–	–
Clarithromycin (15μg)	34.5%	82.8%	–	–	–	–	93.3%	–
Ofloxacin (5μg)	61.2%	6.5%	15.6%	26.9%	3.7%	3.6%	3.2%	10.0%
Gentamicin (10μg)	4.2%	9.4%	36.0%	43.5%	13.8%	13.8%	–	13.6%
Co-trimoxazole (1.25/23.75μg)	4.0%	13.3%	48.1%	70.8%	75.0%	82.1%	90.0%	–
Cefazolin (30μg)	0.0%	100%	32.3%	95.7%	36.4%	93.5%	–	–
Cefuroxime (30μg)	–	100%	24.3%	100%	12.9%	97.1%	–	–
Ceftriaxone (30μg)	–	–	20.0%	96.4%	10.0%	94.1%	7.1%	–
Ceftazidime (30μg)	–	–	2.9%	12.5%	11.1%	67.7%	–	13.6%
Cefotaxime (30μg)	–	–	16.2%	93.1%	13.3%	94.1%	10.0%	–
Cefepime (30μg)	–	–	7.7%	29.6%	0.0%	44.1%	4.2%	4.3%
Vancomycin (30μg)	0.0%	0.0%	–	–	–	–	0.0%	–
Imipenem (10μg)	0.0%	0.0%	0.0%	0.0%	0.0%	0.0%	0.0%	0.0%

*E. coli* exhibited high resistance to piperacillin and ampicillin, but relative low resistance to other antibiotics included ofloxacin, gentamicin and cephalosporins. Similarly, *E. coli* ESBLs had low resistance to ofloxacin and gentamicin, but only it had low resistance to ceftazidime and cefepime in the study.

In the present study, *K. pneumonia* and *K. pneumonia* ESBLs isolates showed similar resistance level to ofloxacin (3.7% vs. 3.6%), gentamicin (13.8% vs. 13.8%) and co-trimoxazole (75.0% vs. 82.1%). *K. pneumonia* displayed low resistance to cephalosporins, but *K. pneumonia* ESBLs exhibited high resistance levels to cephalosporins except for cefepime (44.1%). However, our results showed 24.2% of piperacillin against *K. pneumonia*.

*S. pneumonia* isolates had a resistant rate of 29.0% to penicillin, and a high resistance level of macrolides, including erythromycin (96.7%) and clarithromycin (93.3%). However, our study indicated that *S. pneumonia* isolates had a low resistance level to cephalosporins, such as ceftriaxone (7.1%), cefotaxime (10.0%) and cefepime (4.2%).

Our results showed 21.7% of *P. aeruginosa* were resistant to piperacillin, and had low resistance levels of ceftazidime (13.6%) and cefepime (4.3%) in the study. Our study also exhibited a low resistant rate to other antibiotics, including ofloxaxin (10.0%) and gentamicin (13.6%).

Additionally, we found no bacterial pathogen was resistant to vancomycin and imipenem in the present study.

## Discussion

CA-LRTIs in infants and children continue to be a significant problem worldwide. In recent years, difficulties related to CA-LRTIs treatment in children have greatly increased because of the emergence of resistance to the most widely used antibiotics against some of the bacterial pathogens involved in the development of the disease [14, 20]. Our results showed that MSSA was the most prevalence bacterial pathogen in children with CA-LRTIs, especially in infants less than 1 month. However, it was not in agreement with various studies worldwide that showed *S. pneumonia* to be the most common bacterial agent in children with CA-LRTIs, such as Finland [15], Japan [21] and other cities in mainland China [8–10]. *S. aureus* nasal/nasopharyngeal colonization was common in the less than 6 months, and the positive results might be influenced by specimen from the upper respiratory tract. Our result showed that which bacterial pathogen causing CA-LRTIs was associated with age. However, we need large data to confirm the distribution characteristics among the age groups in the future.

Previous papers ascertained the MRSA cause of CA-LRTIs becoming the major worldwide problem [22]. The resistance rate of MRSA to erythromycin was 81.8%. From 2000 to 2005, Chen et al. [22] found 97% MRSA were resistant to erythromycin in Taiwan. Chen et al. [23] found all the MRSA from infant were resistant to erythromycin during 2008–2009 in Wuhan, China. The decreasing resistance of erythromycin against MRSA may be due to more normative using antibiotics comparing with that in past. MRSA showed resistance rates to ofloxacin (6.5%), gentamicin (9.4%) and co-trimoxazole (13.3%), which was lower than that in USA (ofloxacin of 45%) [15] and Trinidad (gentamicin of 96.8%,co-trimoxazole of 93.1%) [16]. It was associated with limited antibacterial exposure, prescribing and availability in pediatrics in China, in that aminoglycosides and quinolones were inappropriate therapy for pediatric patients [24]. Aminoglycosides might cause hearing loss and quinolones might casuse achondroplasia in children, thus, the two antibiotics were used with caution in China. In early 1990s, the national guidelines were consistent in recommending a macrolide together with a β-lactam in adults with severe CA-LRTIs [25, 26]. Apparently, β-lactam was insignificant to CA-LRTIs with MRSA infections. Limited available data suggested how such community-acquired infections were managed in routine practices. Local and national susceptibility data should be taken into account when making recommendations for empiric therapy. In other parts of China, resistance levels of MSSA to penicillin were substantial. Chen et al. [23] found equivalence to high resistance of 82.9% in Wuhan, China. Hou et al. [27] found 92.5% MSSA were resistant to penicillin in Shenzhen, China. The resistance rate of MSSA to cefazolin was 0.0%, which was in agreement with to that in Shenzhen [27] and Wuhan [23], China. Cefazolin might be recommended the empiric use as first-line treatment to MSSA causing CA-LRTIs in China.

In our study, *S. pneumonia* isolates had a resistant rate of 29.0% to penicillin, which was similar to other surveillance programmers last decade, such as in Taiwan (30%) [22], USA (25%) [14] and Mexico City (37%), but was lower than that in HongKong (56%). With the development of antibiotics, penicillin was rarely used at present in China. Our results showed that high resistance levels to macrolides, including erythromycin (96.7%) and clarithromycin (93.3%). In the early 1990, the US study indicated that CA- *S. pneumonia* had a resistance rate of 10% to erythromycin and clarithromycin [28]. From 2002 to 2005, Matute et al. [29] findings showed CA- *S. pneumonia* had been sensitive to erythromycin and penicillin in León, Nicaragua. Hsueh et al. [30] found 82% CA-*S. pneumonia* were resistant to erythromycin in Taiwan during 1996–1997. In 2012, Pan [31] et al. also found high resistance to erythromycin (98.5%) in Shanghai, China. As observed by various resistance levels, high resistance rates are associated with greater exposure to antibiotics in China [32]. Antibiotics should be used judiciously for reducing antibiotic resistance in China. Fluoroquinolones resistance (ofloxacin, 3.2%) was uncommon among respiratory tract isolates of *S. pneumonia* in the present study, which was in agreement with previous USA report (0.7%) [33]. Our study also identified low resistance to ceftriaxone, which was consistent with that in Shanghai (1%–28.2%) [34, 35]. Generally, the resistance level of *S. pneumonia* against ceftriaxone was consistence with that to cefotaxime. In our study, it might be due to statistical bias.

*K. pneumonia* and *E. coli* were the two Gram-negative bacteria most frequently implicated in causing respiratory tract infections in children in this study, and these results were consistent with other reports from China [36]. They also belong to the family enterobacteriaceae and frequently produce extended-spectrum beta-lactamases. It was important to note that we found no *K. pneumonia* isolate was resistant to cefepime in our study. It might be due to limited isolates. The resistance rate of *E. coli* ELBLs to co-trimoxazole(70.8%) and gentamicin (43.5%) were similar to those reported in Saudi Arabia (co-trimoxazole of 71.1%, gentamicin of 47%) [37]. The resistance of *K. pneumonia* ELBLs to co-trimoxazole(82.1%) and gentamicin (13.8%) were lower than that in Madagascar (co-trimoxazole of 91.3%, gentamicin of 76.1%) [38]. Our results also showed the low resistance level to ofloxacin (26.9%) and cefepine (29.6%). It suggested that these antibiotics were recommended in the clinical treatment. The appropriate drugs against *K. pneumonia* ESBLs were similar with that to *E. coli* ESBLs. But ceftazidime was not recommended empiric use for children with *K. pneumonia* ESBLs infections.

The emergence of carbapenem-resistant *P. aeruginosa* has been an increasing problem in many parts of the world [39, 40]. However, we found 2 strains of carbapenem-resistant *P. aeruginosa* in our study. One report from Taiwan indicated the risk factor of carbapenem-resistant *P. aeruginosa* infections was associated with using fluoroquinolone [41]. It was consistent with the low resistant rate of *P. aeruginosa* to fluoroquinolone (ofloxacin, 9.5%) in our study. Our results also showed *P. aeruginosa* had low resistant rates for ceftazidime (13.0%) and cefepime (4.2%). The resistance levels of *P. aeruginosa* to ceftazidime and cefepime were lower than that in Nepal [42].

Many studies reported that multidrug resistance bacterial infections represented a growing public health problem in children, especially infant under 1-year-old [43]. Limited children with MDR infections were found in the retrospective study. It might be explained by a view that most of patients were from the regular ward. Generally, the patients from Intensive Care Units (ICUs) or Pediatric Intensive Care Units (PICU) were high risk population with MDR infections [43].

There are mainly 2 limitations in this study: Firstly, we did not collect the blood specimens of the patients with CA-LRTIs during the retrospective study, or the result would be more persuasive. Secondly, *mycoplasma pneumoniae* and *chlamydia pneumoniae* infections were also common in the pediatric patients, but patients detected positive for those two pathogens were not enrolled in the study for the two pathogens were identified by other diagnostic methods, and did not detect antibiotic sensitive test.

## Conclusions

*S. aureus*, *E coli* and *K. pneumonia* were the common bacterial isolates recovered from chidren with CA-LTRIs during 2011–2015. Age group of under 1 year old was at a high risk of bacterial infections. Many isolates showed antibiotic resistance level was associated with antibiotic usage in clinic. Increasing surveillance of antibiotic resistance is urgently needed and develops better strategies to cure the antibiotic abuse in China.

## Abbreviations

*A. baumannii*: *Acinetobacter baumannii*
CA-LRTIs: Community acquired lower respiratory tract infections
CLSI: Clinical and Laboratory Standards Institute
*E. coli*: *Escherichia coli*
ESBLs: Producing extended-spectrum beta-lactamases
*H. influenza*: *Haemophilus influenza*
*K. pneumonia*: *Klebsiella pneumonia*
MRSA: Methicillin-Resistant *Staphylococcus aureus*
MSSA: Methicillin-sensitive *Staphylococcus aureus*
*P. aeruginosa*: *Pseudomonas aeruginosa*
*S. aureus*: *Staphylococcus aureus*
*S. pneumonia*: *Streptococcus pneumonia*
